# Pneumococcal colonization impairs mucosal immune responses to live attenuated influenza vaccine

**DOI:** 10.1172/jci.insight.141088

**Published:** 2021-02-22

**Authors:** Beatriz F. Carniel, Fernando Marcon, Jamie Rylance, Esther L. German, Seher Zaidi, Jesus Reiné, Edessa Negera, Elissavet Nikolaou, Sherin Pojar, Carla Solórzano, Andrea M. Collins, Victoria Connor, Debbie Bogaert, Stephen B. Gordon, Helder I. Nakaya, Daniela M. Ferreira, Simon P. Jochems, Elena Mitsi

**Affiliations:** 1 Department of Clinical Sciences, Liverpool School of Tropical Medicine, Liverpool, United Kingdom.; 2 Royal Liverpool and Broadgreen University Hospital, Liverpool, United Kingdom.; 3 Centre for Inflammation Research, Edinburgh Medical School, University of Edinburgh, Edinburgh, United Kingdom.; 4 Department of Paediatric Immunology and Infectious Diseases, University Medical Centre Utrecht, Utrecht, Netherlands.; 5 Malawi-Liverpool Wellcome Trust Clinical Research Programme, College of Medicine, Blantyre, Malawi.; 6 Department of Clinical and Toxicological Analyses, School of Pharmaceutical Sciences, University of São Paulo, São Paolo, Brazil.

**Keywords:** Immunology, Vaccines, Adaptive immunity, Influenza

## Abstract

Influenza virus infections affect millions of people annually, and current available vaccines provide varying rates of protection. However, the way in which the nasal microbiota, particularly established pneumococcal colonization, shape the response to influenza vaccination is not yet fully understood. In this study, we inoculated healthy adults with live *Streptococcus* *pneumoniae* and vaccinated them 3 days later with either tetravalent-inactivated influenza vaccine (TIV) or live attenuated influenza vaccine (LAIV). Vaccine-induced immune responses were assessed in nose, blood, and lung. Nasal pneumococcal colonization had no impact upon TIV-induced antibody responses to influenza, which manifested in all compartments. However, experimentally induced pneumococcal colonization dampened LAIV-mediated mucosal antibody responses, primarily IgA in the nose and IgG in the lung. Pulmonary influenza-specific cellular responses were more apparent in the LAIV group compared with either the TIV or an unvaccinated group. These results indicate that TIV and LAIV elicit differential immunity to adults and that LAIV immunogenicity is diminished by the nasal presence of *S*. *pneumoniae*. Therefore, nasopharyngeal pneumococcal colonization may affect LAIV efficacy.

## Introduction

Each year, 5%–15% of the world’s population will suffer from an influenza infection, with up to 5 million cases of severe disease and 500,000 deaths (1). Influenza viruses have the ability to mutate and hence escape immune defense mechanisms, necessitating annual vaccine updates. These vaccines include the tetravalent inactivated influenza vaccine (TIV) (2), which is given intramuscularly, and the live attenuated influenza vaccine (LAIV) (3), which is administered intranasally. The route of vaccination can trigger distinct immune mechanisms and pathways of protection. For example, TIV is given as an intramuscular injection and induces neutralizing antibodies against strain-specific glycoproteins HA and neuraminidase (NA) (4). By comparison, LAIV is administered intranasally as a cold-adapted vaccine that replicates only in the nasopharynx and mimics natural infection (5). Nasal replication leads to recognition of its pathogen-associated molecular patterns by host pattern recognition receptors, which initiates a cascade of cellular immune responses (6). In mice, LAIV vaccination increases the frequency of CD4^+^ and CD8^+^ T cells in the lung and cytokine production upon influenza restimulation compared with the inactivated virus or no vaccine administration (7–10). Moreover, LAIV seeds the murine lung with both CD4^+^ tissue-resident memory (TRM) and virus-specific CD8^+^ T cells. TRM T cells have been shown to provide long-term cross-strain protection against influenza (7). In humans, the immune responses elicited by LAIV have been found to provide broader clinical protection in children compared with the inactivated influenza vaccines (11). However, the detailed immunological mechanisms of this remain incompletely understood.

Influenza vaccines are reformulated annually to represent circulating strains; however, genomic changes over time (antigenic drift) reduce effectiveness (12). Estimates from WHO suggest that influenza vaccines’ effectiveness rarely exceeds 60% and has fallen below 30% in some years (13, 14). Poor effectiveness of LAIV among youth ages 2–17 in 2014 and 2015 led to the CDC recommending its temporary exclusion from the US national childhood influenza immunization program during the subsequent 2 seasons (15). From 2018, however, no such recommendations have been made. Many underlying causes for this variation have been suggested, including poor matching with circulating strains (12, 16); differential ability of some LAIV types to induce immunity, in particular against H_1_N_1_ strains (15); and the microbial community composition at times of LAIV administration (17).

Despite several reports about the microbiota and their impact on vaccination responses (18–21), including responses to influenza vaccine (20, 22), it remains unclear how the microbiome affects LAIV immunogenicity. In murine models, a prior exposure to *Streptococcus* *pneumoniae* influenza virus potentially compromised long-term antiviral antibody-mediated immunity (23). Colonization of the nasopharynx with pneumococcus is very common during childhood, with a point prevalence of 50% of infants in resource-rich settings and up to 90% in low- and middle-income countries (24). A significant interaction between *S*. *pneumoniae* colonization and influenza vaccination could profoundly impact the utility of vaccination, especially among the poorest groups of the world.

We used an experimental human pneumococcal challenge (EHPC) model (25) to experimentally colonize adults with pneumococcus, who 3 days later received either LAIV (nasal) or TIV (intramuscular). We showed that in humans, LAIV elicited immune responses primarily at mucosal sites of infection — the nose and lung. Interestingly, experimentally induced pneumococcal colonization affected LAIV immunogenicity, dampening the LAIV-mediated nasal and lung immune responses.

## Results

**Figure 1:**
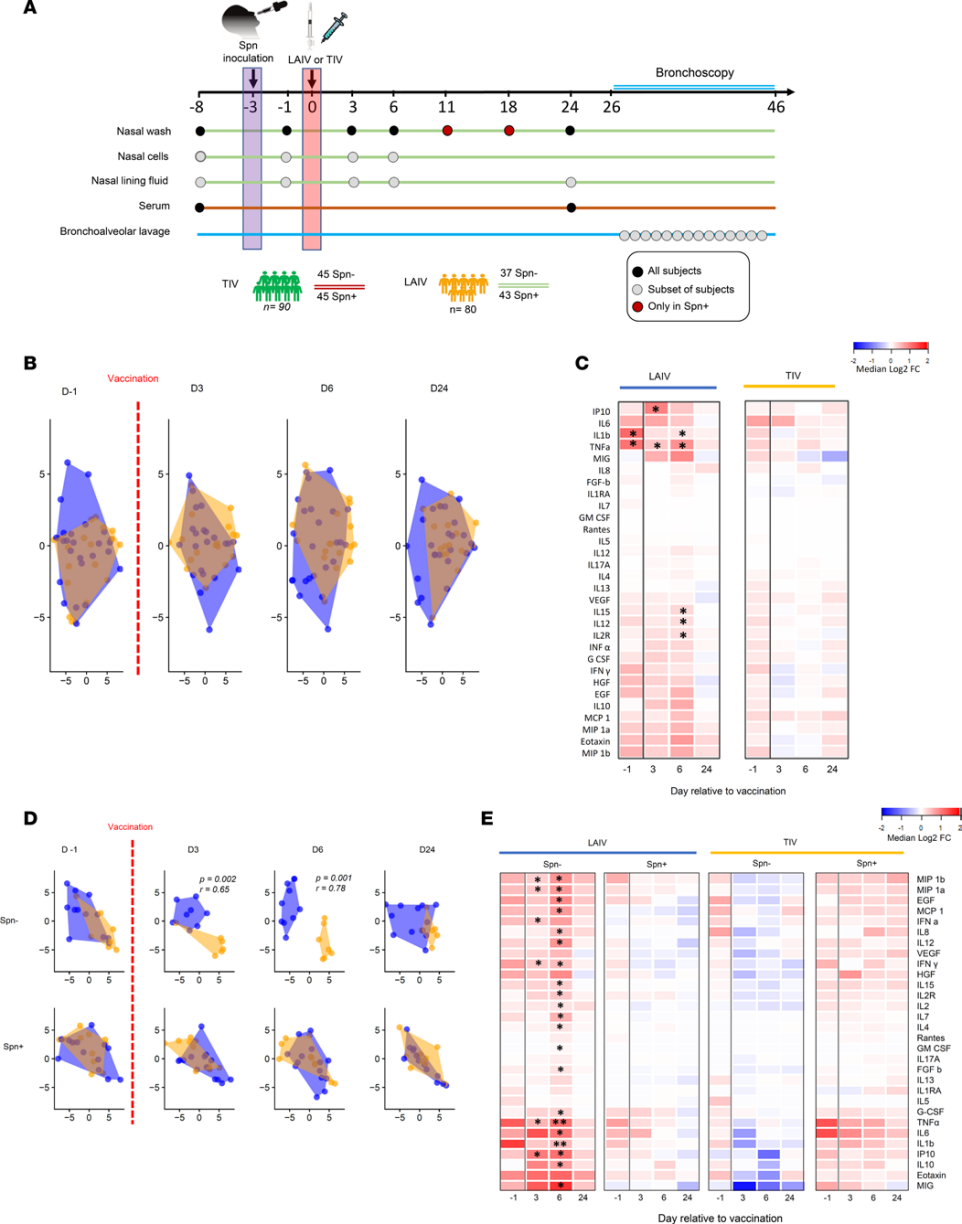
Pneumococcal colonization prevents an acute nasal LAIV-induced proinflammatory response. (**A**) Healthy adults (*n* = 170) 18–48 years of age were recruited and participated in a randomized, controlled clinical trial. Subjects were screened 8 days prevaccination (baseline), followed by challenge with live *Streptococcus* *pneumoniae* (Spn) 3 days before vaccination against influenza (D-3). Then, they received either LAIV or TIV at day 0 (D0). Serum samples were collected at baseline (D-8) and D24. Nasal washes were collected from all volunteers at D-8, D-1, D3, D6, and D24, plus at D11 and D18 for the colonized. Nasal fluid and cells were collected at D-8, D-1, D3, and D6, plus at D24 for nasal fluid only. BAL sample was collected 26–46 days after vaccination. (**B**–**E**) Levels of 30 cytokines were measured in nasal fluid at baseline, 1 day before vaccination (D-1), and 3, 6, and 24 days after vaccination for LAIV/Spn^–^ (LAIV vaccinated/noncolonized, *n* *=* 15), LAIV/Spn^+^ (LAIV vaccinated/colonized, *n* *=* 15), TIV/Spn^–^ (TIV vaccinated/noncolonized, *n* *=* 16) and TIV/Spn^+^ (TIV vaccinated/colonized, *n* *=* 14). (**B** and **D**) Samples were clustered based on fold change (FC) levels to baseline using t-distributed stochastic neighbor embedding for LAIV (blue) or TIV (orange). *R* and *P* values shown for significant time points based on analysis of similarity (anosim), including (FCs) for all cytokines. (**C**) Heatmap showing median log_2_FC to baseline levels at each time point after LAIV or TIV administration, irrespective of colonization status. Upregulation (red) and downregulation (blue) in cytokines’ levels from baseline. (**E**) Heatmap showing median log_2_FC to baseline levels at each time point for the 4 experimental groups, based on stratification by vaccine and colonization status. Statistical comparisons were applied against the baseline sample for each time point in every group independently. **P *<* 0.01, *P *<* 0.05, Wilcoxon’s paired test with Benjamini-Hochberg correction for multiple testing.

We conducted a double-blind randomized controlled clinical trial (26) in which healthy adults (18–48 years of age) were vaccinated with either TIV (*n* = 90) or LAIV (*n* = 80) 3 days after intranasal challenge with live *S*. *pneumoniae* (Figure 1A). To assess and compare the immune responses elicited by influenza vaccination, we analyzed a series of samples in a subset of 40 TIV and 80 LAIV vaccinated subjects. Mucosal samples, including nasal wash, nasal scrapes (epithelial and immune cells), nasal lining fluid, and bronchoalveolar lavage (BAL), as well as serum samples, were collected from the 2 experimental groups and stratified according to vaccination and pneumococcal carriage status: (a) TIV vaccinated non–*S*. *pneumoniae* colonized (TIV/*S*. *pneumoniae^–^*, *n* = 21), (b) TIV vaccinated *S*. *pneumoniae*–colonized (TIV/*S*. *pneumoniae^+^*, *n* *=* 19), (c) LAIV vaccinated non–*S*. *pneumoniae* colonized (LAIV/*S*. *pneumoniae^–^*, *n* = 37), and (d) LAIV vaccinated *S*. *pneumoniae*-colonized (LAIV/*S*. *pneumoniae^+^*, *n* *=* 43). For the assessment of lung immune responses, we included a nonvaccinated cohort as control (*n* *=* 20, 10 *S*. *pneumoniae^–^* and 10 *S*. *pneumoniae^+^*, respectively), since we were only able to sample the human lung after challenge/vaccination and not at baseline.

### *S. pneumoniae* colonization prevents an acute nasal proinflammatory response upon LAIV administration.

Vaccine-induced inflammatory responses in the nasal mucosa were assessed by measuring levels of 30 cytokines in the nasal fluid at baseline, at day 1 (2 days after *S*. *pneumoniae* challenge but 1 day before vaccination), and at 3, 6, and 24 days after vaccination. LAIV administration induced a mild proinflammatory response, which resembled TIV based on similarity analysis (Figure 1B). In particular, only IFN-γ–inducible protein 10 (IP-10) and TNF-α were significantly increased (*P* *<* 0.05 by Wilcoxon’s test with Benjamini-Hochberg adjustment for multiple testing correction) at 3 days after LAIV. At day 6, TNF-α remained at increased levels compared with prechallenge baseline (day 8 time point), and levels of 4 more cytokines (IL-1b, IL-12, IL-15, and IL-2R) had a transient induction at this time point (Figure 1C). No other cytokine was significantly induced in either the LAIV or TIV group at any time point.

To investigate whether colonization of the nasopharynx with *S*. *pneumoniae* prior to transient LAIV infection would alter the LAIV-mediated immunogenicity, we stratified the groups according to volunteers’ colonization status and assessed the cytokine profile in the 4 experimental groups. LAIV induced a transient but robust proinflammatory response only in the absence of nasal pneumococcal colonization (Figure 1D). In particular, macrophage inflammatory protein 1α (MIP-1α), MIP-1β, IFN-γ, IFN-α, IP-10, and TNF-α were significantly increased from prechallenge baseline at 3 days after LAIV in the noncolonized group (Figure 1E). At 6 days after LAIV, 21 out of 30 measured cytokines were significantly increased in this group (Figure 1E). No other cytokine was significantly induced in any of the 4 groups at any time point.

### LAIV increases the frequency of influenza-specific TNF-α– and IFN-γ–producing CD4^+^ and TRM CD4^+^ T cells in the lung.

Data from animal models suggest that LAIV, but not TIV, induces protective cellular responses in the lung (27, 28). To assess influenza vaccination-induced cellular responses in the human lung, BAL cells were stimulated with influenza antigens. T cell subsets (CD4^+^, CD8^+^, and TCR-γδ^+^) were immunophenotyped, and cytokine production was measured by intracellular cytokine staining in order to determine the frequency of IFN-γ–, IL-17A–, and TNF-α–producing influenza-specific T cells (Supplemental Figure 1; supplemental material available online with this article; https://doi.org/10.1172/jci.insight.141088DS1). Frequencies of total CD4^+^, CD8^+^, and TCR-γδ^+^ T cells were not affected by vaccination status (Supplemental Figure 2). Furthermore, we investigated the presence of TRM T cell responses to influenza, using the extracellular markers CD69, CD103, and CD49a. Because more than one-third of CD4^+^CD69^+^ cells, commonly defined as TRM (29), did not express the additional resident memory markers CD103 and CD49a, we defined TRM only as CD69^+^ (Supplemental Figure 3). In contrast, nearly all CD8^+^CD69^+^ cells also expressed CD103 and CD49a.

**Figure 2:**
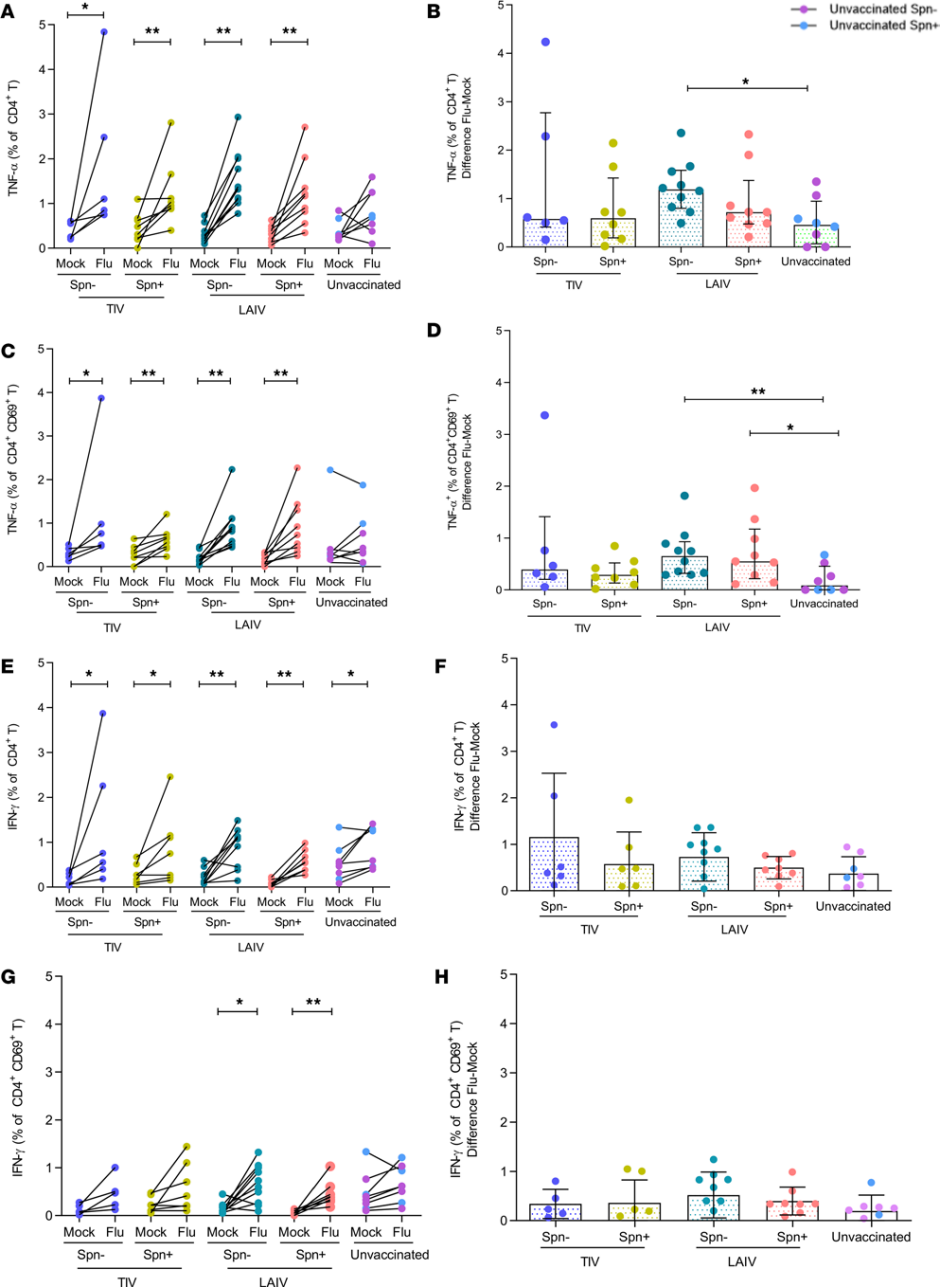
LAIV increases frequency of influenza-specific TNF-α– and IFN-γ–producing CD4^+^ and TRM CD4^+^ T cells in the lung. Frequencies of cytokine-producing CD4^+^ and TRM CD4^+^ T cells were measured in human BAL samples by intracellular staining flow cytometry analysis with and without (mock) in vitro influenza antigen stimulation. Volunteers were divided by vaccine and colonization status in TIV/Spn^–^ (*n* *=* 6), TIV/Spn^+^ (*n* *=* 8), LAIV/Spn^–^ (*n* *=* 10), LAIV/Spn^+^ (*n* *=* 9), and unvaccinated (*n* *=* 8, 3 Spn^–^ and 5 Spn^+^) groups. (**A**) Production of TNF-α by total CD4^+^ T cells in each group (paired unstimulated [mock] and stimulated condition [flu]). (**B**) Influenza-specific production of TNF-α by total CD4^+^ T cells (difference between influenza-stimulated and unstimulated) in each group. (**C**) Production of TNF-α by CD4^+^CD69^+^ T cells in each group. (**D**) Production of influenza-specific TNF-α by CD4^+^CD69^+^ T cells in each group. (**E**) Production of IFN- γ by total CD4^+^ T cells in each group. (**F**) Production of influenza-specific IFN-γ by CD4^+^ T cells in each group. (**G**) Production of IFN-γ by CD4^+^CD69^+^ T cells and (**H**) influenza-specific IFN-γ by CD4^+^CD69^+^ T cells in each group. Each individual dot represents a single volunteer, and the conditions from 1 individual are connected. Medians with IQR are depicted for influenza-specific responses (**B**, **D**, **F**, and **H**). *P *<* 0.05, **P *<* 0.01 by Wilcoxon’s test for comparisons within the same group and by Mann-Whitney *U* test for between-group comparisons.

CD4^+^ TNF-α production upon influenza stimulation was observed in both TIV and LAIV recipients, regardless of colonization status, but not in unvaccinated individuals (Figure 2, A and B). However, levels of influenza-specific TNF-α were significantly increased in the LAIV/*S*. *pneumoniae^–^* group compared with the unvaccinated group (median 2.6-fold increase, *P* *=* 0.015; Figure 2B).

Following stimulation with influenza antigens, CD4^+^ TRM T cells produced TNF-α in all vaccinated groups but not in the unvaccinated group (Figure 2C). The induction of TNF-α–producing CD4^+^ TRM T cells following stimulation did not significantly differ between TIV and LAIV, but it was more pronounced in the LAIV group, in both the *S*. *pneumoniae^–^* and *S*. *pneumoniae^+^* groups compared with the unvaccinated group (7.7-FC, *P* *=* 0.004; and 6.5-FC, *P* = 0.024 unvaccinated, respectively, Figure 2D).

We also assessed IFN-γ production by total CD4^+^ and TRM CD4^+^ T cells residing in the human lung. IFN-γ production by total CD4^+^ T cells was observed in all groups upon stimulation, including the unvaccinated group (Figure 2E). The levels of IFN-γ–producing CD4^+^ T cells were not different when comparing vaccinated and unvaccinated groups. The induction of IFN-γ–producing CD4^+^ TRM T cells, however, was greater in the LAIV vaccinated group (Figure 2F). In contrast to total CD4^+^ T cells, stimulation of TRM T cells of unvaccinated individuals did not elicit an IFN-γ response (Figure 2G). Pneumococcal colonization status did not affect the levels of either influenza-specific IFN-γ–producing CD4^+^ or TRM IFN-γ–producing CD4^+^ T cells (Figure 2, F–H).

Furthermore, the proportion of IL-17A–producing CD4^+^ T or CD4^+^ TRM T cells was not affected by vaccination with either TIV or LAIV (Supplemental Figure 4, A and B). The frequency of regulatory CD4^+^ T cells was also measured in the lung, with such cells showing increased levels in BAL samples of LAIV/*S*. *pneumoniae^–^* compared with unvaccinated individuals (mean 1.5-fold increase) (*P* = 0.039; Supplemental Figure 5).

### LAIV increases the frequency of influenza-specific TNF-α–producing CD8^+^ and TRM CD8^+^ T cells in the lungs.

**Figure 3:**
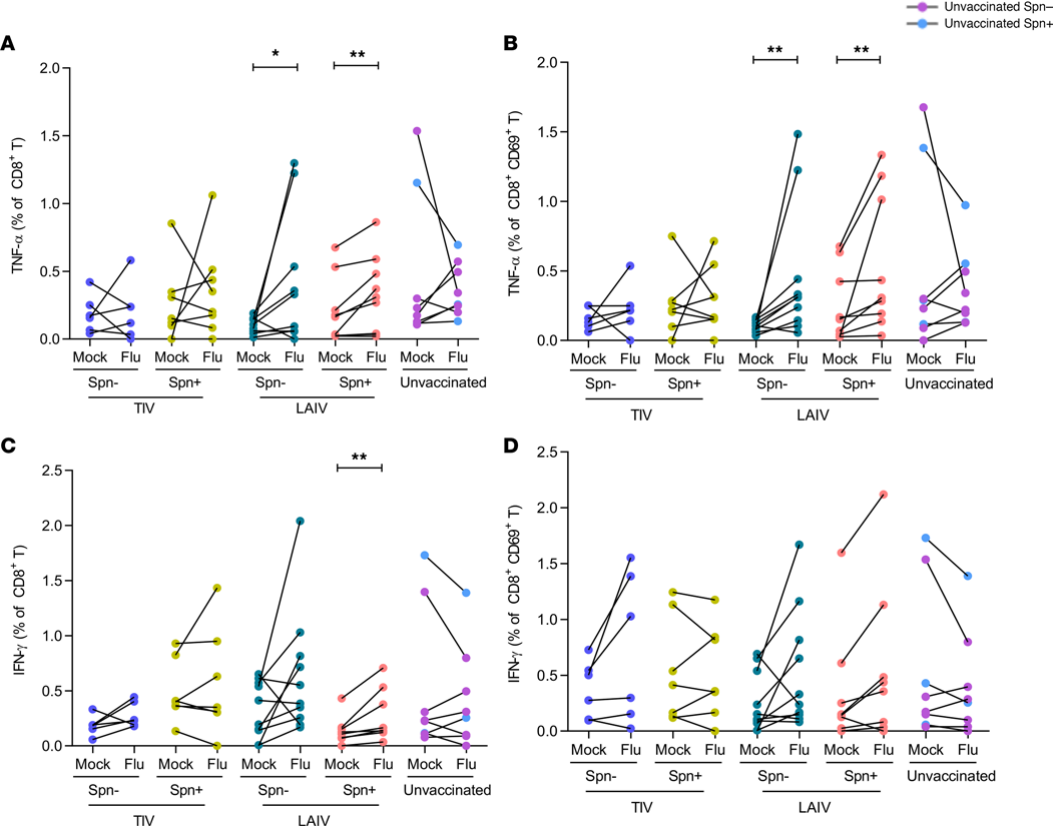
LAIV increases frequency of influenza-specific TNF-α–producing CD8^+^ and TRM CD8^+^ T cells in the lungs. Frequencies of cytokine-producing CD8^+^ T cells were measured in human BAL samples by intracellular staining flow cytometry analysis following stimulation with influenza antigens or nonstimulation (mock) in each group. Volunteers were divided by vaccine and colonization status in TIV/Spn^–^ (*n* = 6), TIV/Spn^+^ (*n* = 8), LAIV/Spn^–^ (*n* = 10), LAIV/Spn^+^ (*n* = 9), and unvaccinated (*n* = 8, 3 Spn^–^ and 5 Spn^+^) groups. Production of TNF-α by (**A**) total CD8^+^ T cells and (**B**) TRM CD8^+^ T cells in each group (paired unstimulated [mock] and stimulated condition [flu]). Production of IFN-γ production by (**C**) total CD8^+^ T cells and (**D**) TRM CD8^+^ T cells in each group. Each individual dot represents a single volunteer, and the conditions per individual are connected. *P *<* 0.05, **P *<* 0.01 by Wilcoxon’s test.

In vitro restimulation with influenza induced increased production of TNF-α by CD8^+^ T cells in the LAIV but not the TIV or unvaccinated group (Figure 3). When volunteers were stratified based on colonization status, LAIV/*S*. *pneumoniae^–^* had a median 2.3-fold increase of TNF-α–producing CD8^+^ T cells after stimulation compared with the nonstimulated condition (*P* *=* 0.03). LAIV/*S*. *pneumoniae^+^* group had a similar induction on this type of cellular response (median 1.9-fold increase, *P* *=* 0.007; Figure 3A). Similarly, TNF-α production by TRM CD8^+^ cells was observed only in the LAIV vaccinated group, increased by median 3.1-FC (*P* *=* 0.006) and 2.1-FC (*P* *=* 0.004) in the LAIV/*S*. *pneumoniae^–^* and LAIV/*S*. *pneumoniae^+^* groups, respectively (Figure 3B).

IFN-γ responses by lung CD8^+^ T cells after stimulation were confined in the LAIV group. Although both LAIV/*S*. *pneumoniae^–^* and LAIV/*S*. *pneumoniae^+^* groups had the same levels of induction (1.5-fold increase) in the proportion of IFN-γ–producing CD8^+^ T cells after stimulation (Figure 3C), this effect was statistically significant only in the LAIV/*S*. *pneumoniae^+^* group due to the lower variance within the group (Figure 3C). TIV and control groups had overall no increase in the proportion of IFN-γ–producing CD8^+^ T cells after stimulation with influenza antigens. In addition, IFN-γ production by lung TRM CD8^+^ T cells was not significantly altered after stimulation in any of the groups (Figure 3D).

Stimulation did not elicit production of IL-17A–producing CD8^+^ T cells, except for IL-17A production by TRM CD8^+^ T cells in the *S*. *pneumoniae*–colonized group (2.6-fold increase, *P* = 0.008; Supplemental Figure 4, C and D).

### LAIV increases frequency of influenza-specific IFN-γ–producing TCR-γδ^+^ T cells in the lungs of noncolonized individuals.

**Figure 4:**
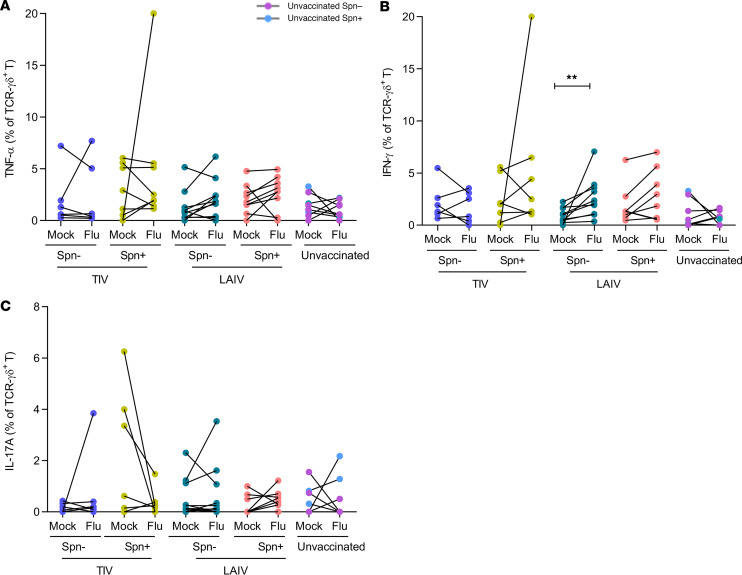
LAIV increases frequency of IFN-γ–producing influenza-specific TCR-γδ^+^ in the lungs of noncolonized individuals. Frequency of cytokine-producing TCR-γδ^+^ T cells was measured in human BAL samples by intracellular staining flow cytometry analysis after in vitro stimulation with influenza antigens or nonstimulation (mock). Volunteers were divided by vaccine and colonization status in TIV/Spn^–^ (*n* = 6), TIV/Spn^+^ (*n* = 8), LAIV/Spn^–^ (*n* = 10), LAIV/Spn^+^ (*n* = 9), and unvaccinated (*n* = 8, 3 Spn^–^ and 5 Spn^+^) groups. Production of (**A**) TNF-α, (**B**) IFN-γ, and (**C**) IL-17A by lung TCR-γδ T cells. Individual dot represents a single volunteer, and the conditions per individual are connected. **P *<* 0.01 by Wilcoxon’s test.

TCR-γδ cells, a subset of specialized innate-like T cells that can exert effector functions immediately upon activation, play an important role in pulmonary infection (30, 31). Therefore, we assessed whether TCR-γδ responses to influenza antigens were induced following vaccination. Although no significant increase in TNF-α–producing TCR-γδ^+^ was observed in any of the groups (Figure 4A), the proportion of IFN-γ–producing TCR-γδ^+^ was significantly greater in the LAIV/group (median 2.9-fold increase upon stimulation compared with the unstimulated condition, *P* = 0.002, Figure 4B). None of the other vaccinated or unvaccinated groups showed a significant induction of IFN-γ production. Similar to the other T cell subsets, IL-17A–producing TCR-γδ^+^ cells did not significantly increase after stimulation with influenza antigens (Figure 4C).

### *S. pneumoniae* colonization impairs nasal IgA induction following LAIV but does not alter responses to TIV.

**Figure 5:**
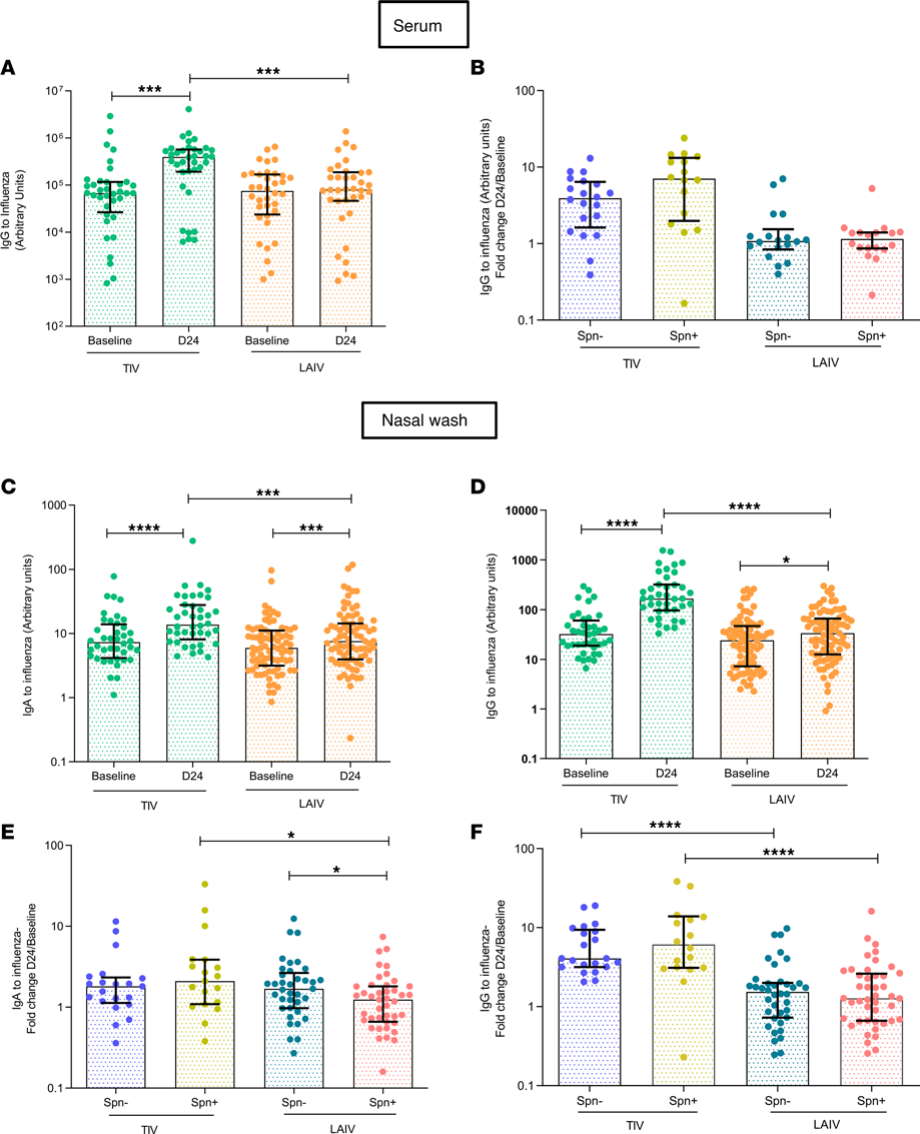
LAIV vaccination increases levels of antibody against influenza in serum and nasal wash, with impaired nasal production caused by *S. pneumoniae* colonization. (**A**) IgG titers to influenza, measured by ELISA, in serum of LAIV (*n* *=* *36*) and TIV (*n* *=* *36*) vaccinated subjects at baseline (8 days prevaccination) and D24 (24 days after vaccination). (**B**) FC (D24/baseline) of paired IgG titers to influenza in serum following TIV or LAIV vaccination. TIV/Spn^–^ (*n* = 20), TIV/Spn^+^ (*n* = 16), LAIV/Spn^–^ (*n* *=* 18), and LAIV/Spn^+^ (*n* = 18). (**C**) IgA and (**D**) IgG titers against influenza measured by ELISA in nasal wash of TIV (*n* *=* 40) and LAIV (*n* = 80) vaccinated subjects at baseline (8 days before vaccination) and D24 (24 days after vaccination). (**E**) FC (D24/baseline) of paired IgA and (**F**) IgG titers against influenza in nasal wash following vaccination with TIV/Spn^–^ (*n* = 21), TIV/Spn^+^ (*n* = 19), LAIV/Spn^–^ (*n* = 37), and LAIV/Spn^+^ (*n* = 43). Medians with IQR are shown. *P *<* 0.05, ***P *<* 0.001, ****P *<* 0.0001 by Wilcoxon’s test for comparisons within the same group and by Mann-Whitney *U* test for comparisons between groups.

In addition to cellular responses, we sought to assess humoral responses elicited by TIV and LAIV vaccination both systemically and at the mucosal sites (nasal and lung). In serum samples, IgG levels against influenza antigens were measured at baseline (prior to bacterial challenge and influenza immunization) and at day 24 after vaccination. TIV induced a 5.9-fold increase (*P* *<* 0.0001) of influenza-specific IgG, whereas LAIV intranasal administration did not confer increase of sera IgG levels (Figure 5A). Prior colonization of the nasopharynx with *S*. *pneumoniae* did not alter influenza-specific IgG levels induced in response to either vaccine (Figure 5B).

To assess antibody responses at the nasal mucosa, we measured influenza-specific IgA and IgG levels in nasal wash samples at baseline and 24 days following influenza immunization and described the kinetics of influenza-specific IgM at baseline, D3, D6, D11, and D24 in both vaccine groups. TIV induced a median 2.2- and a 5.2-fold increase in influenza virus-specific IgA and IgG levels, respectively, 24 days after vaccination (Figure 5, C–F). LAIV also elicited an IgA and IgG antibody response, though both (IgA median 1.3-fold increase and IgG median 1.4-fold increase) were lower compared with those induced by TIV (Figure 5, C–F). In addition, TIV induced an earlier and stronger IgM response in nasal mucosa compared with LAIV. The median levels of influenza-specific IgM titers in nasal lavage were 1.71, 2.74, and 1.94 times higher compared with baseline levels at D6, D11, and D24, respectively, in the TIV group, whereas in the LAIV group they differed statistically significantly from baseline only at day 24 after vaccine administration (median 1.22-fold increase from baseline, Supplemental Figure 6A).

LAIV-mediated immunogenicity at the nasal mucosa was also dependent on *S*. *pneumoniae* colonization, as observed for the lung cellular responses. Experimentally induced colonization of the nasopharynx with *S*. *pneumoniae* affected IgA titers, but neither IgG nor IgM, in the LAIV group (Figure 5, E and F, and Supplemental Figure 6C). At day 24 after vaccination, the LAIV/*S*. *pneumoniae^–^* group had significantly greater levels of IgA against influenza circulating in the nasal lumen, compared with the LAIV/*S*. *pneumoniae^+^* counterparts (LAIV/*S*. *pneumoniae^–^* median = 1.69, IQR: 0.98–2.65 vs. LAIV/*S*. *pneumoniae^+^* median = 1.24, IQR: 0.66–1.81) (*P* = 0.02; Figure 5E). *S*. *pneumoniae* colonization did not alter humoral responses to influenza in the TIV group for any antibody isotype (Figure 5, E and F, and Supplemental Figure 6B).

### IgG but not IgA is induced by influenza vaccines in the lung, with LAIV-mediated responses being impaired by pneumococcal colonization.

**Figure 6:**
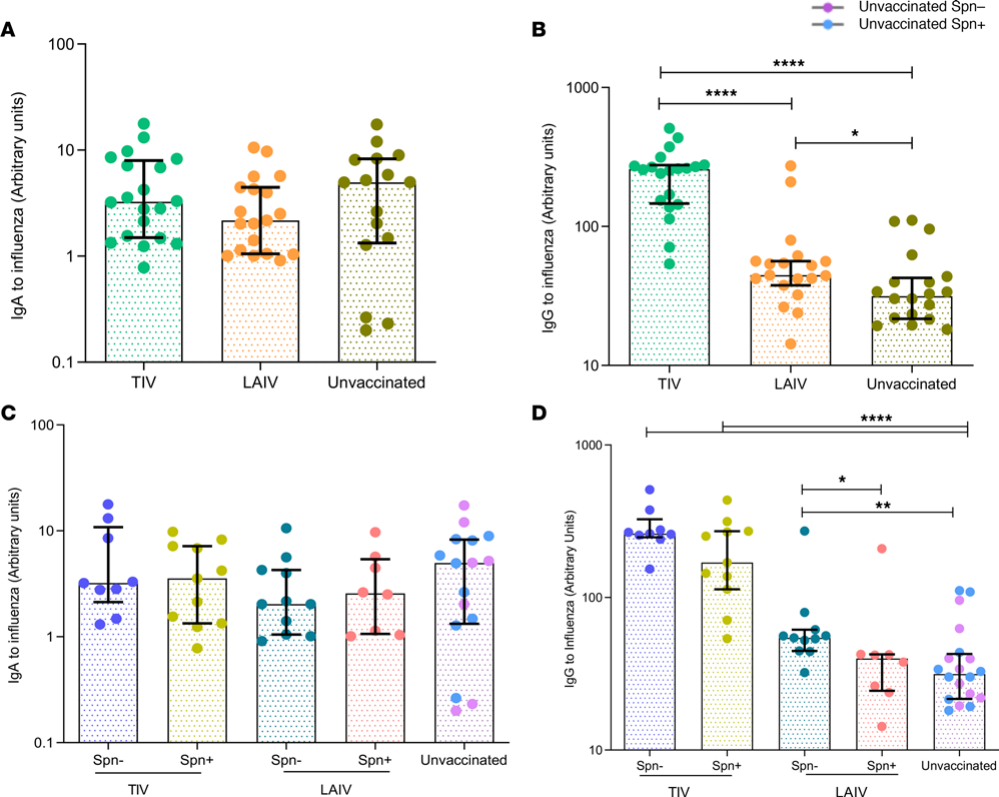
IgG but not IgA is induced by influenza vaccines in the lung, with LAIV responses being reduced during *S. pneumoniae* colonization. (**A** and **B**) IgA and IgG titers against influenza for TIV (*n* = 20), LAIV (*n* = 19) vaccinated subjects and unvaccinated (*n* = 20) was measured by ELISA in BAL fluid. (**C** and **D**) IgA and IgG titers grouped based on vaccination and colonization status, as TIV/Spn^–^ (*n* = 9), TIV/Spn^+^ (*n* = 11), LAIV/Spn^–^ (*n* = 11), LAIV/Spn^+^ (*n* = 8), and unvaccinated (*n* = 20). Medians with IQR are shown. *P *<* 0.05, **P *<* 0.01, ****P *<* 0.0001 by Wilcoxon’s test for comparisons within the same group and by Mann-Whitney test *U* for comparisons between groups.

Humoral responses in the lung following TIV or LAIV vaccination were assessed in BAL samples collected between 26 and 46 days after influenza vaccination (Figure 6). Owing to the single time point sampling of the lung, 20 unvaccinated subjects (10 *S*. *pneumoniae*–colonized and 10 noncolonized) were used as a control group.

IgA against influenza levels in the lung did not differ between the TIV, LAIV, and control groups (Figure 6A). In terms of IgG levels, TIV induced a high IgG response (median 5.8-fold increase compared with control) (*P* *<* 0.0001), whereas LAIV conferred a modest IgG induction (median 1.6-FC compared with control) (*P* = 0.028; Figure 6B). TIV-elicited influenza-specific IgG levels were 3.7 times greater than LAIV-induced responses in the pulmonary mucosa (Figure 6B). Despite the late time point after vaccination, IgM against influenza was detectable in the lung, and higher titers were measured in the TIV (3.3-fold increase compared with control) (*P* = 0.0003) than in the LAIV vaccinated subjects (1.81-fold increase compared with control) (*P* = 0.022; Supplemental Figure 6D).

IgA levels were not significantly increased in the lung by vaccination and therefore not affected by *S*. *pneumoniae* colonization (Figure 6C). Similar to IgA, IgM responses in the lung did not differ between *S*. *pneumoniae–*colonized and noncolonized subjects (Supplemental Figure 6E). However, *S*. *pneumoniae* colonization affected IgG titers in the LAIV vaccinated group but not in the TIV group. IgG against influenza was higher in the LAIV/*S*. *pneumoniae^–^* group compared with the control group (1.73-fold increase, *P* *=* 0.006), whereas the LAIV/*S*. *pneumoniae^+^* group did not differ from the control (Figure 6D).

#### TLR priming by *S. pneumoniae* and increased type I IFN gene expression profile soon after nasal colonization establishment.

**Figure 7:**
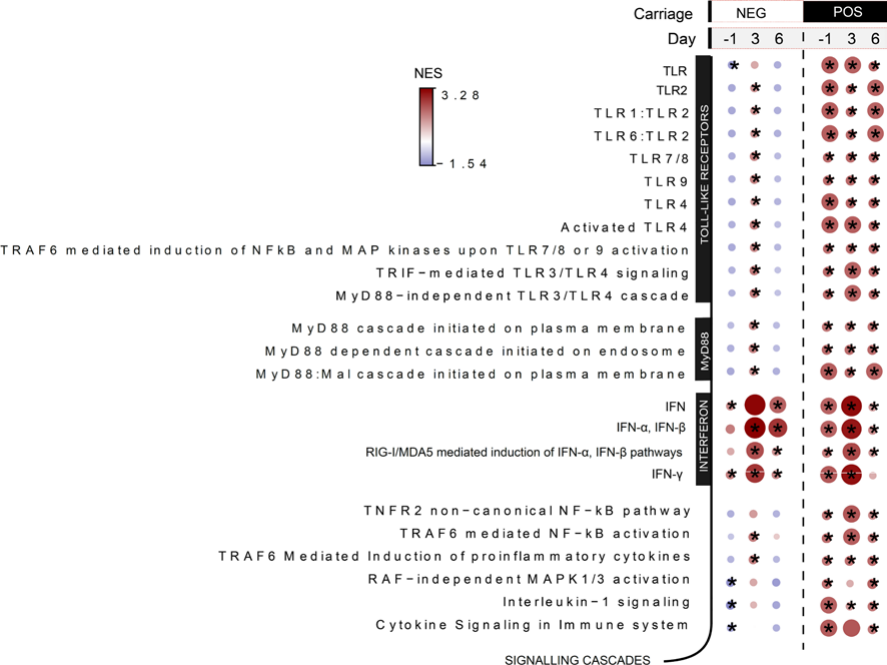
TLR priming by *S. pneumoniae* and increased type I IFN gene expression profile soon after nasal colonization. Selected pathways after gene set enrichment analysis for LAIV/Spn^–^ (*n* = 11) and LAIV/Spn^+^ (*n* = 9) groups at D-1, D3, and D6 in relation to LAIV administration applied on log_2_FCs (baseline/pre-Spn inoculation-normalized values). Normalized enrichment score (NES) is presented in gradient color. Red shades indicate pathways overrepresented, whereas blue shades depict the underrepresented pathways at each time point in relation to baseline (prior pneumococcal inoculation). *P *<* 0.05 by Wilcoxon’s paired test corrected by multiple-comparison testing (Benjamini-Hochberg).

To identify molecular signatures associated with reduced LAIV-mediated immunogenicity and impaired inflammatory responses owing to established pneumococcal nasopharyngeal colonization, we performed host RNA-Seq on nasal cells at baseline, day 1 (before vaccination), and at 3 and 6 days after vaccination. Two days after the bacterial challenge but prior to influenza vaccination, the LAIV/*S*. *pneumoniae^+^* group showed enrichment in genes related to TLR signaling, including TLR2, TLR4, and TLR9 (Figure 7). As expected, gene enrichment in TLR was not observed in the LAIV/*S*. *pneumoniae^–^* group at the same time point after inoculation. Additionally, the LAIV/*S*. *pneumoniae^+^* group exhibited enrichment in IFN-α/β, IFN-γ genes and RIG-I/MDA5–mediated induction of IFN-α/β pathways (Figure 7). The upregulation of these pathways suggests that pneumococcal colonized volunteers had increased antiviral responses the day before the LAIV administration, a molecular profile that is likely to interfere with the influenza virus replication cycle in the nasopharynx.

To further investigate this observation, influenza RNA was quantified in unconcentrated nasal washes collected at 3 days after vaccination in the LAIV group. Only one-fourth of the LAIV vaccinated group had detectable levels of influenza viral RNA in the nose 3 days after vaccination, with no statistically significant differences in the levels of influenza viral RNA (Supplemental Figure 7A) or in the percentage of shedders (Ct < 40) between the *S*. *pneumoniae* colonized (23.1%, 9/39) and noncolonized (27.5%, 11/40; Supplemental Table 1). As expected, levels of influenza-specific IgA, following LAIV vaccination, were greater (2.5-FC) in the nasal mucosa of volunteers with detectable viral influenza replication (Supplemental Figure 7B). In contrast, raised influenza-specific IgG levels following vaccination did not differ between shedders and nonshedders (Supplemental Figure 7C).

## Discussion

We investigated the cellular and humoral immune responses elicited by TIV and LAIV, focusing on respiratory mucosa, and assessed whether colonization of the nasopharynx with *S*. *pneumoniae* influences vaccine immunogenicity. In agreement with previous studies (3), TIV vaccination induced high systemic and mucosal antibody responses, whereas LAIV elicited both mucosal (mainly IgA) influenza virus-specific antibodies and cell-mediated immune responses. Interestingly, experimentally induced pneumococcal colonization of the nasopharynx impaired host immunity to LAIV but did not alter TIV-induced responses. Antecedent pneumococcal colonization was also associated with weakened acute nasal proinflammatory responses after LAIV vaccination.

In the lungs, LAIV-induced cellular responses were heightened and markedly increased from those induced by TIV. LAIV nasal administration led to increased levels of TNF-α– and IFN-γ–producing CD4^+^ T cells, including TRM T cells, as well as TNF-α–producing CD8^+^ T cells, upon in vitro stimulation. Interestingly, we observed that influenza-specific CD4^+^ T cell lung responses were more pronounced in individuals not colonized with *S*. *pneumoniae* at the time of vaccination, suggesting increased immunogenicity of LAIV in the absence of pneumococcal colonization. Similarly, there was a higher proportion of IFN-γ–producing TCR-γδ^+^ T cells in the noncolonized LAIV recipients. Moreover, LAIV was associated with increased frequencies of lung Tregs but only in the absence of nasal *S*. *pneumoniae* colonization.

Humoral responses were highly induced by TIV, whereas LAIV conferred an overall modest antibody induction. Systemically, TIV elicited influenza virus-specific IgG responses, which were not observed in the LAIV vaccinated arm. In the nose, TIV conferred predominantly IgG induction, whereas LAIV was mainly associated with high levels of IgA. Colonization of the nasopharynx with *S*. *pneumoniae* at the time of LAIV administration impaired the induction of mucosal IgA to influenza in the nose and IgG in the lung. The modulatory effect of *S*. *pneumoniae* on adaptive immune responses to influenza virus has been previously reported in a murine coinfection model, highlighting the importance of current pathogen exposure, which can critically affect the generation of protective antiviral antibodies and subsequently reduce influenza vaccination efficacy (23).

The protection provided by LAIV relies on a transient viral replication in the nasopharynx to induce sufficient antibody levels against influenza, which we observed with increased IgA induction in shedders compared with nonshedders. LAIV in adults, unlike children, does not confer superior protection compared with TIV (32). An explanation consistent with the hypothesis is that lifelong accumulation of influenza immunity through natural exposure and previous vaccinations can prevent the nasal replication of the attenuated virus and shorten the viral replication cycle (33). Here, 25.3% of young adults vaccinated with LAIV shed attenuated influenza virus (either influenza A or B), in contrast to the much higher shedding rates observed in 2- to 5-year-old children in other studies (34). Taking into consideration virus neutralization by preexisting antibodies against influenza due to lifetime exposure or a shortened virus replication cycle (33), increased influenza shedding has to be detected in the LAIV cohort in the first 2 days after the vaccine administration. Consequently, LAIV may elicit less potent responses in adults compared with children; thus, any extrapolation from findings in adults to children, the target population for this vaccine, must be done with caution.

Children display high rates of *S*. *pneumoniae* colonization (35, 36). Our finding that concurrent *S*. *pneumoniae* colonization could inhibit LAIV-induced immune responses is another variable that should be taken into account when evaluating LAIV efficacy. This phenomenon could explain the lack of LAIV efficacy reported in Senegal (37), as *S*. *pneumoniae* colonization rates are higher in low-income countries (38). The impaired LAIV-induced immunity during established *S*. *pneumoniae* colonization was associated with a lack of a proinflammatory response in the nasal mucosa following LAIV vaccination. An explanation for this is that *S*. *pneumoniae* colonization affects local immune and epithelial cell responses upon LAIV vaccination, which could diminish immune cell infiltration and antigen-presenting cell activation, impacting the downstream memory responses (39, 40). For instance, lack or reduced production of IFNs by activated nasal cells after vaccination may affect innate immune responses to LAIV, by impairing NK and macrophage activation in the nasal mucosa and potentially DC migration and differentiation. Such an impaired innate immune response would also translate to reduced antigen presentation and subsequently affect the adaptive immune responses.

It is also possible that *S*. *pneumoniae* colonization interferes with the viral replication cycle (41, 42), through stimulation of TLR. A number of studies have reported the broad contribution of TLR2 to the antiviral IFN response by indirectly governing the production of IFNs induced by other TLRs, as well as downstream of the cytosolic Rig-I–like receptors (43, 44). In an infant mouse influenza A–*S*. *pneumoniae* coinfection model, mice deficient for TLR2 showed decreased expression of IFN-α and higher viral titers than WT animals, with this great viral burden correlating with heightened inflammation (45). In our study, *S*. *pneumoniae–*colonized volunteers upregulated genes involved in TLR2, RIG-I/MDA5-mediated induction of IFN-α/β, and IFN-α/β pathways before exposure to LAIV and exhibited impaired inflammatory responses after vaccination. A strong induction of TLR and IFN pathways by *S*. *pneumoniae* colonization may result in quick viral sensing and resolution of viral infection. Despite these observations, any alteration of viral replication cycle mediated by *S*. *pneumoniae* colonization was limited by the late time point of viral quantification at 3 days after LAIV administration. An alternative hypothesis of the curtailed viral shedding in the LAIV/*S*. *pneumoniae^+^* group would be the inhibitory effect of pneumococcal neuraminidases, particularly NanA, on influenza virus attachment to the epithelium, as shown in an infant mouse model of the *S*. *pneumoniae*–influenza A virus coinfection model (46). In light of these observations, it would also be interesting to investigate to what extent symptoms and inflammation caused by WT influenza viruses are altered by concurrent *S*. *pneumoniae^+^* colonization in humans.

Ideally, an effective and broadly protective influenza vaccine should induce both humoral and cellular immunity. Whereas antibody responses to influenza show some degree of strain cross-reactivity (47, 48), they are insufficient to provide heterosubtypic, cross-strain influenza protection (49, 50). Recent data from longitudinal cohort studies of naturally acquired infection have highlighted the potential of T cells as key players in mediating heterosubtypic immunity in humans (51, 52). We observed that even in the absence of vaccination, healthy adults showed CD4^+^ T cell responses to influenza stimulation, which likely reflects their lifelong exposure to influenza viruses. The use of purified, adjuvanted antigen influenza vaccine (TIV) as the stimulus to measure cellular responses in vitro would possibly lead to greater T cell responses. Our results demonstrated that LAIV induced influenza-specific cytokine-producing CD8^+^ and CD4^+^ T cells, including TRM T cells in the lung. Such cells are important during influenza infection in protection of mucosal barrier tissues against pathogen challenge by producing chemokines for cell recruitment (53). It has been shown that TRM T cells provide superior protection against influenza infection when compared with circulating T cells (54). By seeding the lungs with these cells, it is possible to establish long-term heterosubtypic protection to influenza (55, 56).

We have also demonstrated that, in volunteers who were not colonized by *S*. *pneumoniae*, LAIV increased levels of Tregs in the lung compared with unvaccinated individuals. CD4^+^ Tregs contribute to homeostasis of the immune system, controlling infection by respiratory viruses and avoiding secondary bacterial infection (57). As a result of recurrent exposure to virus and bacteria, CD4^+^ Tregs increase in frequency with age (58). For this reason, our findings in adults might underestimate the effect of LAIV on frequency of Tregs in the lungs of children.

Although LAIV- and TIV-mediated responses were assessed in the context of a randomized clinical trial, the study was limited by evaluation of a single pneumococcal serotype in healthy adults likely to have neutralizing influenza antibodies and cross-reactive T cells. In addition, we evaluated pneumococcal and live attenuated virus vaccine interaction during the early stages of pneumococcal colonization, when host responses to bacterial exposure may be higher compared with later time points. It is possible that this short-term window may not reflect accurately the overall effect of a colonization episode on LAIV immune responses. Any LAIV effect in children may be different owing to lower antibody titers against influenza, higher natural rates of pneumococcal colonization, and higher levels of inflammation compared with young adults (59). A future pediatric study, whereby colonization status is assessed before LAIV administration and correlated with immune responses to the vaccine, would provide important insights into the magnitude of pneumococcal effect on vaccine immunogenicity in this population.

In conclusion, using a controlled human model in which pneumococcal infection occurred at a known time relative to vaccination, we were able to highlight differences in immunogenicity between LAIV and TIV at relevant mucosal sites. Moreover, we identified *S*. *pneumoniae* colonization as an important variable in LAIV-induced immunity.

## Methods

### Study design.

Adult volunteers were enrolled in the parent LAIV clinical trial study (REC 14/NW/1460; ref. 26). Exclusion criteria included a prior history of influenza or pneumococcal vaccination, clinically confirmed pneumococcal disease in the preceding 2 years, pregnancy, close contact with individuals at increased risk for pneumococcal disease (children under 5, immunosuppressed people, and elderly people), recent febrile illness, current or recent use of antibiotics, or immune-modulating medication. Participants were inoculated with 80,000 CFU per nostril of serotype 6B as previously described (25). All volunteers received an influenza vaccination 3 days after pneumococcal inoculation. The LAIV group (*n* = 80) received the LAIV (2016/2017 Fluenz Tetra), whereas the TIV group (*n* = 90) received the TIV (2016/2017 Fluarix Tetra). These 2 vaccine formulations had the same combination of influenza A and influenza B strains. The overall carriage rates did not differ between the LAIV and TIV groups as measured by conventional microbiology (37/80 [46.3%] vs. 45/90 [50.0%], respectively).

For investigation of immune responses, samples of nasal wash, nasal lining fluid, nasal cells, BAL, and serum were collected from volunteers at specific time points, processed, and frozen for future analysis (Supplemental Figure 1). For comparisons within the lung data sets, BAL fluid and lung lymphocytes from an unvaccinated EHPC group (*n* *=* 20, 10 *S*. *pneumoniae^–^* and 10 *S*. *pneumoniae^+^*) were used as a control.

### Detection of *S pneumoniae* colonization.

To detect bacteria in the nasopharynx, nasal wash sample plates were examined by classical microbiology for presence of *S*. *pneumoniae* as previously described (60, 61). Colonized individuals were defined as anyone who had a positive nasal wash sample at any time point following inoculation.

### BAL analysis.

A BAL sample was collected at the end of the trial, between 26 and 46 days after vaccination. Bronchoscopy was performed using topical anesthesia, and BAL was collected as described previously (62). Briefly, a total of 200 mL warm 0.9% saline was instilled and retrieved from a subsegmental bronchus of the right middle lobe by hand suction. BAL was placed into sterile tubes on ice and processed as previously described (62). Next, the BAL sample was filtered to remove mucus and centrifuged at 400*g* for 10 minutes. BAL cells were resuspended in RPMI medium with antibiotic mixture (Penicillin-Streptomycin-Neomycin, Thermo Fisher Scientific). Cells were plated in a 24-well plate (Greiner Bio-One) to allow macrophages to adhere for 4 hours at 37°C, 5% CO_2_. Nonadherent BAL cells were collected, washed, centrifuged at 200*g* for 10 minutes, and resuspended in RPMI medium prior to stimulation.

### Intracellular cytokine staining.

Nonadherent BAL cells were counted and incubated at 1 × 10^6^ cells/mL in medium with RPMI medium FBS (10% heat inactivated, Thermo Fisher Scientific) and antibiotic mixture (Penicillin-Streptomycin-Neomycin) at 37°C. Samples were stimulated with 1.2 μg/mL influenza antigens (TIV, 2016/2017) or left unstimulated as negative control and incubated for 2 hours. Then, 1000× diluted GolgiPlug (BD Biosciences) was added, and cells were cultured for an additional 16 hours.

After 16 hours, the cells were washed with 3 mL PBS, resuspended, and stained with Violet Viability dye (LIVE/DEAD Fixable Dead Cell stain kit, Thermo Fisher Scientific). After 15 minutes, the cells were stained with the surface markers CD3-APC-H7 (clone SK7) and TCR-γδ–PECy7 (clone 11F2) from BD Biosciences and CD4–PerCP5.5 (clone SK3), CD8–AF700 (clone SK1), CD69–BV650 (clone FN5O), CD25-PE.TxsRed (clone M-A251), CD103–BV605 (clone Ber-ACT8), and CD49a-APC (clone TS2/7) (all from BioLegend) and incubated for 15 minutes. Cells were fixed and permeabilized using the Foxp3/Transcription Factor Staining Buffer Set (eBioscience, Thermo Fisher Scientific) as per the manufacturer’s instructions. Cells were then stained with intracellular markers FOXP3-FITC (clone 259D), IFN-γ–PE (clone 4S.B3), and TNF-α–BV711 (clone MAb11) (all from BioLegend) and IL-10–BV786 (clone JES3-9D7) IL-17A–BV510 (clone N49-653) from BD Biosciences. After 30 minutes, samples were washed with 3 mL PBS and resuspended in 200 μL PBS and acquired on a BD LSR flow cytometer (Becton Dickinson). Flow cytometry data were analyzed using cell analysis software version 10 (FlowJo, LLC).

### Quantitative reverse-transcription PCR.

Quantitative reverse-transcription PCR was used to quantify nasal virus shedding in volunteers vaccinated with LAIV. RNA was isolated (RNeasy kit; Qiagen) from nasal wash fluid, following generation of cDNA (high-capacity RT kit; Applied Biosystems, Thermo Fisher Scientific) for use in quantitative PCR (SYBR Green PCR master mix; Applied Biosystems, Thermo Fisher Scientific). Samples were tested using primers, probes, and PCR assay conditions specific for human influenza virus A and B (63). Results were analyzed using the cycle threshold (2 ΔΔCt) method by comparison with GAPDH transcription.

### ELISA.

ELISA was used to quantify levels of IgG and IgA antibodies against influenza in the serum, as well as IgG, IgA, and IgM in nasal wash and BAL supernatant of volunteers vaccinated with TIV or LAIV or unvaccinated. Pooled serum of 7 TIV vaccinated volunteers was heat-inactivated (at 56°C for 30 minutes) and used as standard in both total IgA and IgG against influenza ELISA. Antibody levels were expressed in arbitrary units relative to this standard curve. For IgG detection, an initial standard dilution of 1:4000 was used, whereas for IgA it was diluted 1:40.

Briefly, 96-well plates (Nunc) were coated with 100 μL 0.2 μg/mL TIV in PBS overnight at room temperature. After the overnight incubation, plates were washed following blocking with 100 μL PBS with 1% BSA for 1 hour at room temperature. Plates were washed, and then samples were added in duplicates and incubated for 2 more hours at room temperature. Each wash consisted of washing the plate 3 times with PBS with 0.005% Tween 20 (MilliporeSigma).

For detection of IgG, IgA, and IgM, a 1:5000, 1:4000, and 1:2000 dilution of anti-human IgG (MilliporeSigma, A9544), anti-human IgA (MilliporeSigma, A9669), and anti-human IgM (MilliporeSigma, A3437), respectively, was made using 0.1% BSA and 100 μL added to each well after washing and incubated at room temperature for 1 hour.

Next, plates were washed, and 100 μL p-Nitrophenyl Phosphate (MilliporeSigma) was added to the wells. The OD of each well was measured at 405 nm using a FLUOstar Omega ELISA microplate reader (BMG Labtech), the average blank corrected value was calculated for each sample, and the data analyzed using Omega Analysis (BMG Labtech).

### Luminex analysis of nasal lining fluid.

Nasal lining fluid was collected using nasosorption filters as previously described (64) and stored at –80°C until analysis. Prior to analysis, cytokines were eluted from stored filters using 100 μL assay diluent buffer (Thermo Fisher Scientific) by centrifugation. The eluate was cleared by further centrifugation at 16,000*g* for 10 minutes. Samples were acquired on an LX200 using a 30-plex magnetic human Luminex cytokine kit (Thermo Fisher Scientific), and results were analyzed with xPonent3.1 software (Luminex) following the manufacturer’s instructions. Samples were analyzed in duplicates, and cytokines with a coefficient of variation of more than 25% for a given sample were excluded from further analysis.

### RNA extraction and sequencing.

Nasal cells were collected in RNALater (Thermo Fisher Scientific) at –80°C until extraction. RNA extraction was performed using the RNEasy Micro Kit (Qiagen) with on-column DNA digestion. Extracted RNA was quantified using a Qubit (Thermo Fisher Scientific). Sample integrity assessment (Bioanalyzer, Agilent), library preparation, and RNA-Seq (Illumina Hiseq4000, 20 million reads, 100 paired-end reads) were performed at the Beijing Genome Institute.

### RNA-Seq analysis.

Quality control of raw sequencing data was done using fastQC. Mapping to a human reference genome assembly (GRCh38) was done using STAR 2.5.0a (65). Read counts from the resulting BAM alignment files were obtained with featureCounts using a GTF gene annotation from the Ensembl database (66, 67). The R/Bioconductor package DESeq2 was used to identify differentially expressed genes among the samples, after removing absent features (0 counts) (68). Genes with an FDR value of less than 0.1 and an absolute FC of more than 1.5 (baseline-normalized values) were identified as differentially expressed. For each time point comparison, gene set enrichment analysis was performed using the fgsea R package. Genes with Ensembl IDs were transformed into gene symbols by the biomaRt package (69) and ordered by their log FC values. Preranked genes and Reactome gene sets from Enrichr (70) were provided to fgsea, with remaining default parameters. To identify significant common pathways between all comparisons, pathways with a *P* value below a threshold of 0.05 for at least 1 comparison were selected and clustered based on the NES with hierarchical clustering. Correlation plots were generated to display the NES values using the corrplot package.

### Data availability.

Raw RNA-Seq data have been deposited in the National Center for Biotechnology Information’s Gene Expression Omnibus repository, accession number GSE164649. All other underlying data are provided in the manuscript.

### Statistics.

All sampling, processing, and data analysis were performed while blinded to vaccination group to not bias results. Nonparametric tests were used for statistical analysis where number of samples was insufficient for a normal distribution of results. Statistics were calculated in GraphPad Prism software, version 6.0 and 7.0 for Windows, and R statistical software (R Foundation for Statistical Computing). A *P* value of less than or equal to 0.05 was considered significant. Benjamini-Hochberg multiple correction was performed in R on both 30-plex cytokine data and RNA-Seq data analysis.

### Study approval.

Ethical approval was given by the North West-Liverpool East Research Ethics Committee (REC) reference number 14/NW/1460. The trial was registered on EudraCT, Protocol 2014-004634-26 (NCT ID: NCT03502291). All volunteers gave written informed consent, and research was conducted in compliance with all relevant ethical regulations. BAL samples of the control (nonvaccinated cohort) were collected at part of a separate EHPC clinical trial (REC 15/NW/0931).

## Author contributions

DMF, SPJ, and EM conceived and designed the study. BFC, ELG, J Reine, E Negera, E Nikolaou, SP, and DB acquired the data. BFC, FM, J Reine, E Negera, HN, SP, HN, DMF, SPJ, and EM analyzed and interpreted the data. J Rylance, SZ, AMC, SC, and VC assisted in clinical procedures and recruitment. BFC wrote the first draft of the paper. All authors commented on and approved the paper.

## Supplementary Material

Supplemental data

## Version 1 01/26/2021

In-Press Preview

## Version 2 02/22/2021

Electronic publication
