# Supplementary material for: Pneumococcal colonization impairs mucosal immune responses to live attenuated influenza vaccine
Source: JCI Insight. 2021 Feb 22;6(4):e141088. doi: 10.1172/jci.insight.141088 (PMC7934923; doi:10.1172/jci.insight.141088)
Supplement: Supplemental data [file jciinsight-6-141088-s107.pdf]

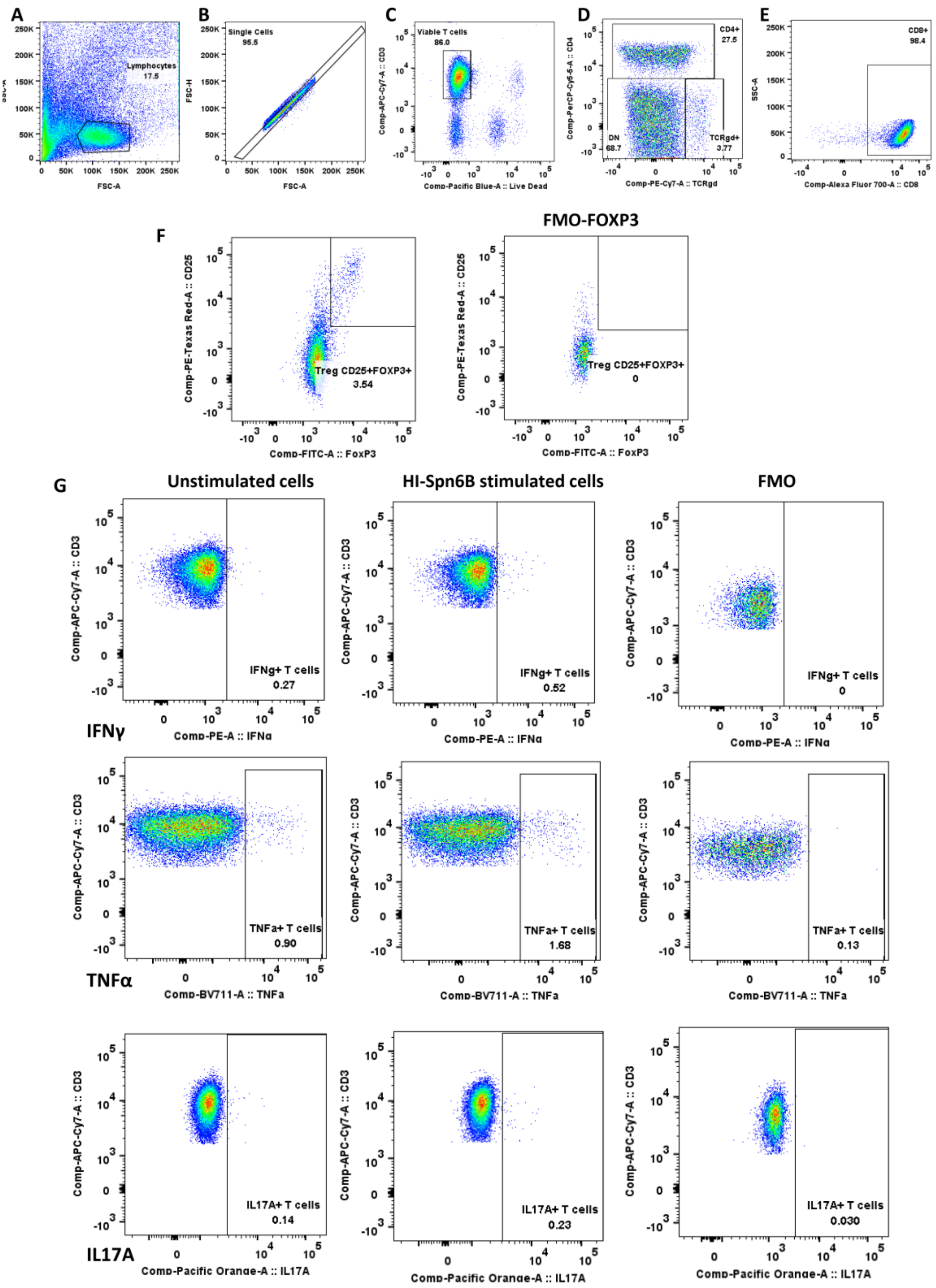

Supplemental Figure 1. Representative gating strategy to identify T cells and cytokine production by flow cytometry using intracellular staining. The following gating strategy was used: **(A)** FSC-A/SSC-A to **(B)** FSC-H/FSC-A (in order to exclude doublets) to **(C)** CD3/Viability to **(D)** CD4/TCR-γδ to **(E)** SSC-A/CD8. **(F)** The markers CD25/FOXP3 ($CD25^{hi}$ and $FOXP3^{+}$) were used to assess the frequency of regulatory T cells. To assess cellular production of **(G)** IFN-γ, (H) IL-17A, (I) TNF-α, cells were stained by intracellular staining after overnight mock or influenza-stimulation. Fluorescence Minus One (FMO) controls were used to verify flow cytometric data.

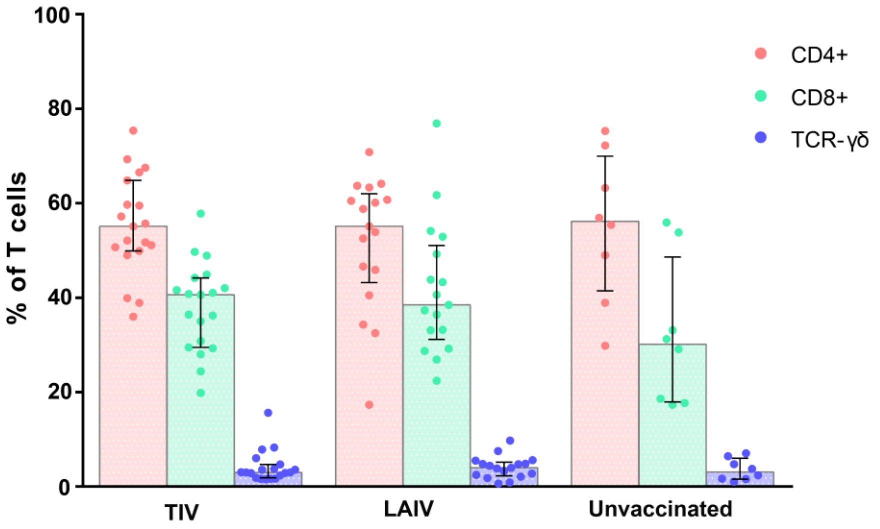

Supplemental Figure 2. Neither LAIV nor TIV affect frequencies of total CD4^{+}, CD8^{+} and TCR-γδ T cell subsets in the lung. Subset frequencies among viable T cells were measured after an overnight incubation of isolated BAL cells. Bars depict the median proportion of T-cell subsets among total T-cells for TIV/Spn- (n=9), TIV/Spn+ (n=11), LAIV/Spn- (n=11) and LAIV/Spn+ (n=9). Also, unvaccinated Spn- (n=3) and unvaccinated Spn+ (n=5).

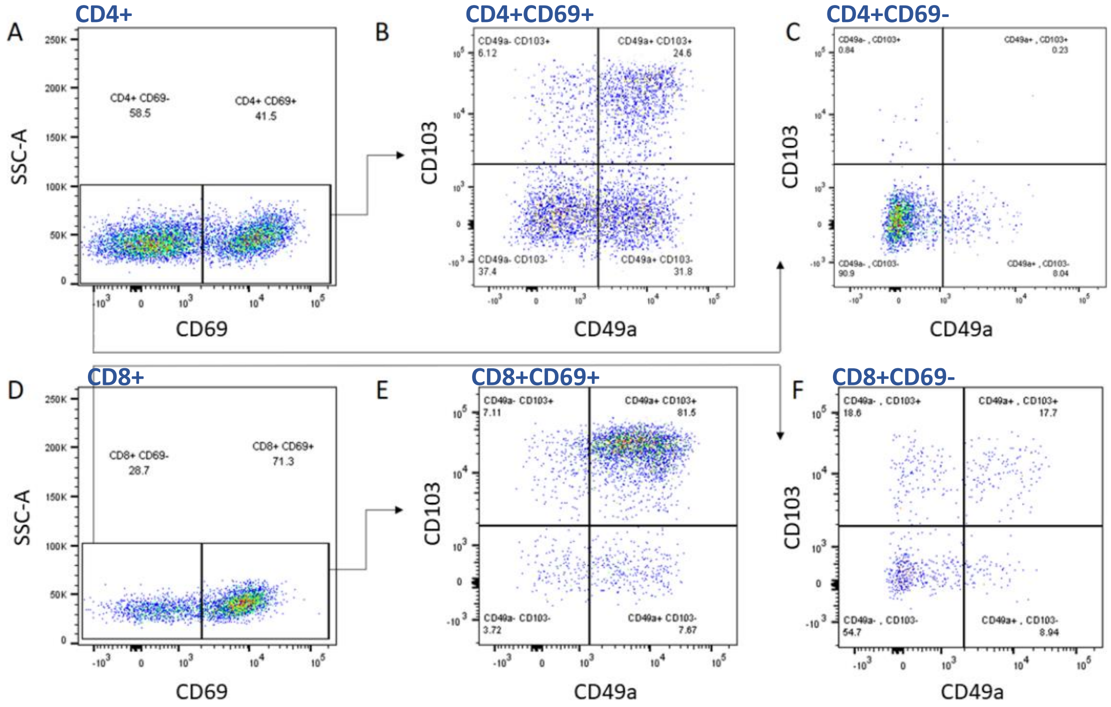

Supplemental Figure 3. Representative plots of tissue resident memory T-cells identified by flow cytometry. The markers CD69, CD103 and CD49a were used to assess the frequency of tissue resident memory cells in human BAL. **(A)** CD4^{+} T-cells gated into CD69 negative and positive cells. CD103 and CD49 marker expression are shown for **(B)** CD4^{+} CD69^{+} T cells and **(C)** CD4^{+} CD69^{-} T cells. **(D)** CD8^{+} T cells gated into CD69 negative and positive cells. CD103 and CD49 marker expression are shown for **(E)** CD8^{+} CD69^{+} T cells and **(F)** CD8^{+} CD69^{-} T cells.

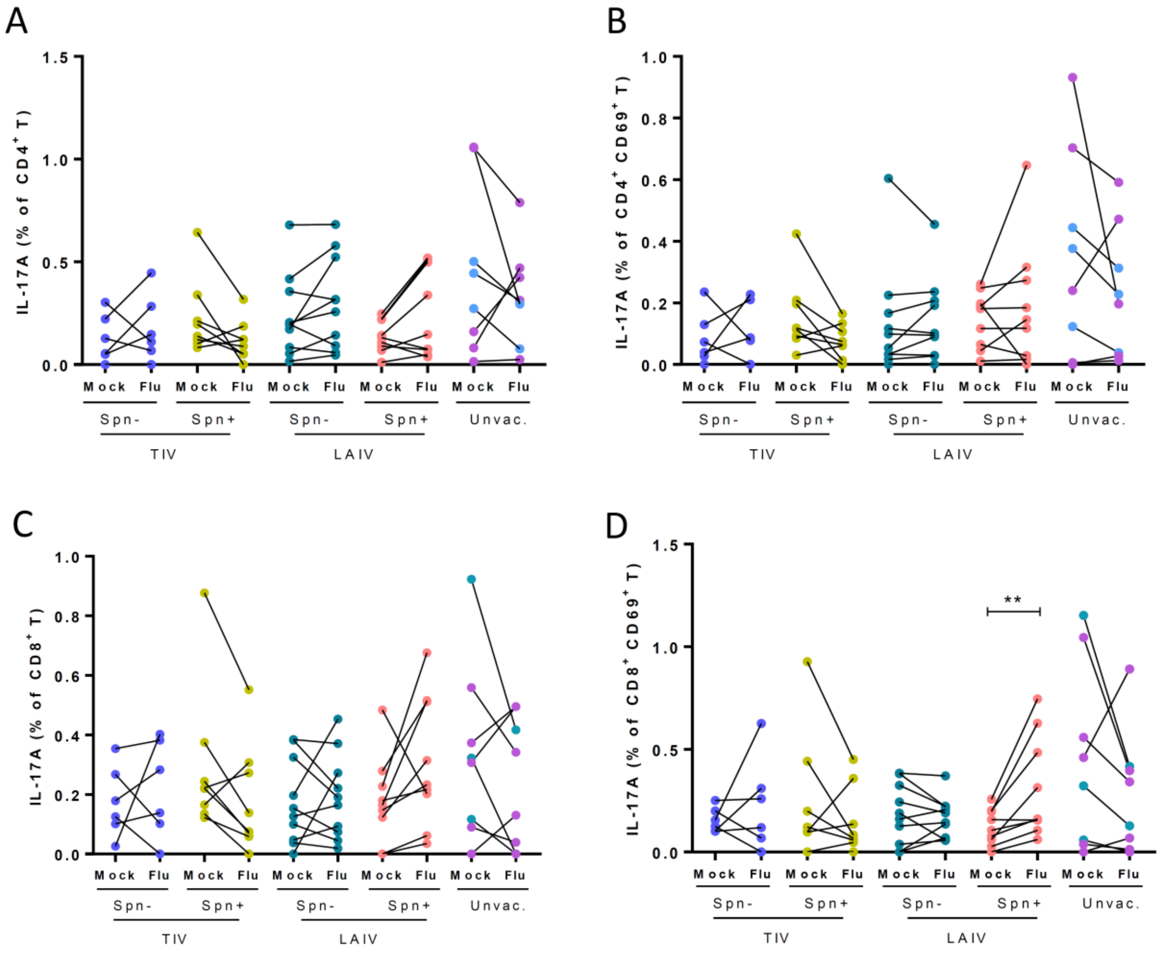

Supplemental Figure 4. LAIV and TIV do not increase the frequency of influenza-specific, IL-17A-producing T-cells in the lung. The frequency of cytokine-producing cells was measured by intracellular staining flow cytometry analysis after stimulation with influenza antigens or unstimulated for TIV Spn- (TIV vaccinated/non-colonized, n=8), TIV Spn+ (TIV vaccinated/colonized, n=6), LAIV Spn- (LAIV vaccinated/non-colonized, n=10), LAIV Spn+ (LAIV vaccinated/colonized, n=9), unvaccinated Spn- (n=3), unvaccinated Spn+ (n=5). IL-17A production in **(A)** total CD4$^+$ T-cells, **(B)** CD4$^+$ CD69$^+$ T-cells, **(C)** total CD8$^+$ T-cells and **(D)** CD8$^+$ CD69$^+$ T-cells. Each individual dot represents a single volunteer and the conditions from one individual are connected. **$p < 0.01$. The unstimulated and influenza antigen-stimulated responses were compared within each group by Wilcoxon test. Influenza-specific responses (influenza-stimulated - unstimulated) were compared between the groups using Mann-Whitney test.

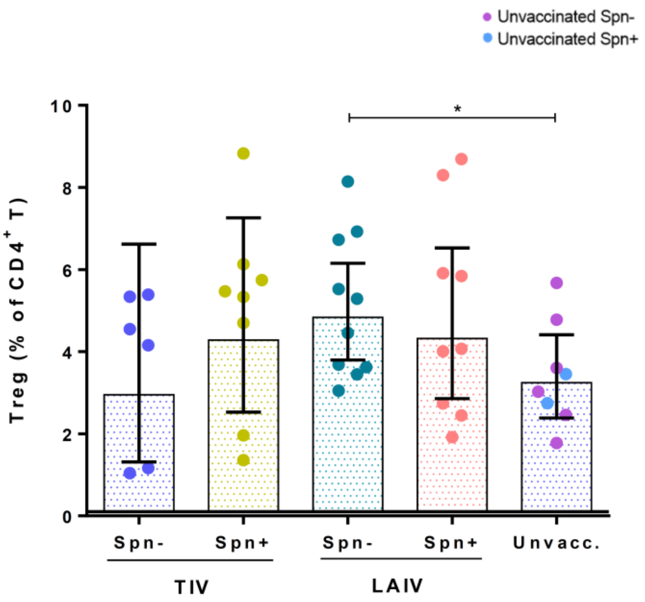

Supplemental Figure 5. **LAIV increases frequency of CD4$^+$ regulatory T-cells in the lung of Spn non-colonized individuals.** Frequency of unstimulated CD4$^+$ T-regs (CD3$^+$ CD4$^+$ CD25$^+$ FOXP3$^+$) was measured by flow cytometry in human BAL samples from TIV/Spn- (n=6), TIV/Spn- (n=8), LAIV/Spn- (n=10), LAIV/Spn+ (n=9) and unvaccinated (n=8, 3 Spn- and 5 Spn+). Each individual dot represents a single volunteer and geometric means with 95% CI are shown. *$p < 0.05$ by unpaired t test.

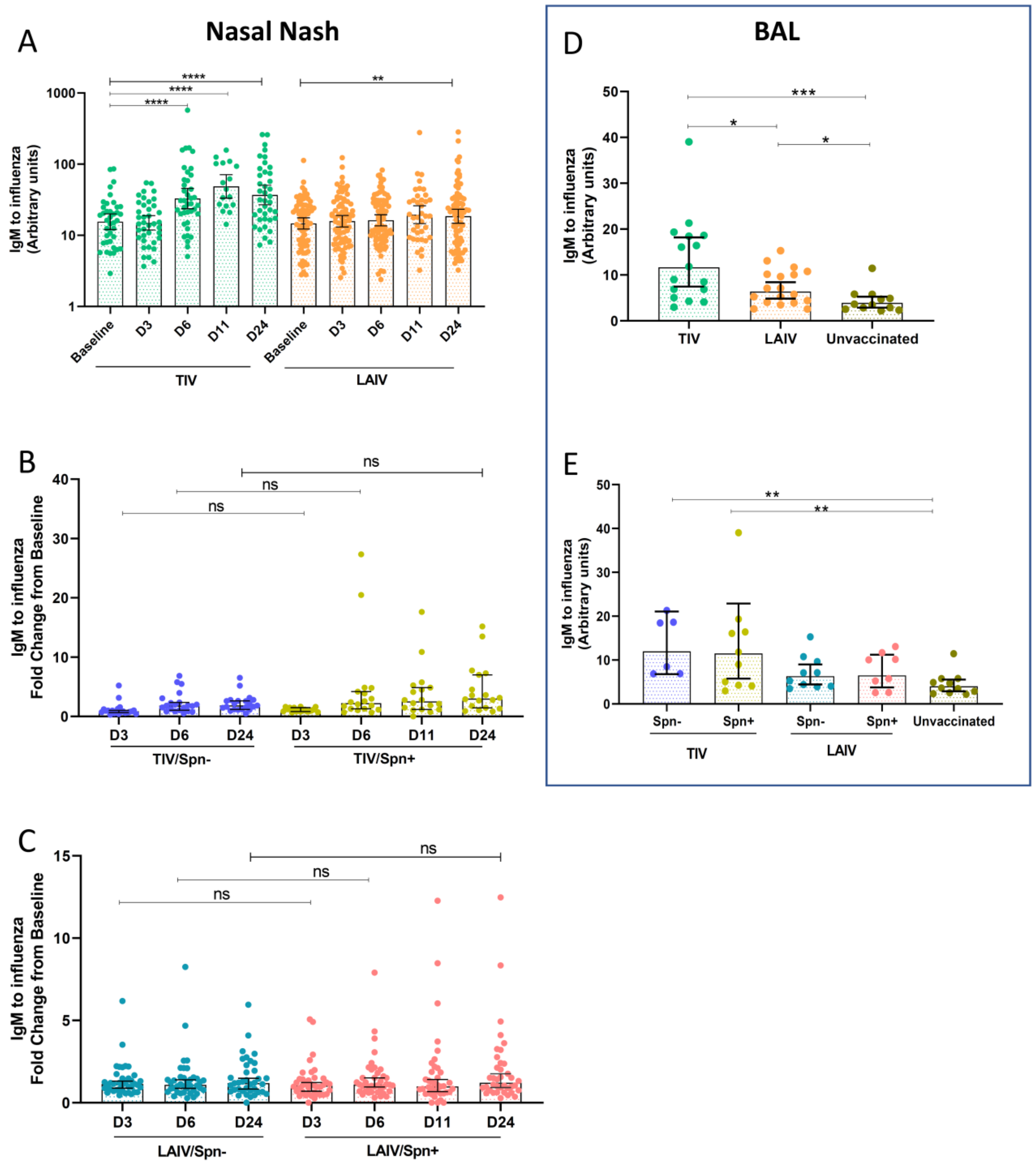

Supplementary figure 6: TIV induces robust IgM responses at both nasal and lung mucosa. A) Kinetics of influenza-specific IgM titres measured by ELISA, in nasal wash of TIV (n=40) and LAIV (n=80) vaccinated subjects at baseline, D3, D6, D11 and D24 post vaccination. **B)** Fold change from baseline of paired IgM titres to influenza at D3, D6, D11 (only Spn+) and D24 in nasal wash of TIV/Spn- (*n=21*) and TIV/Spn+ (*n=19*). **C)** Fold change from baseline of paired IgM titres to influenza at D3, D6, D11 (only Spn+) and D24 in nasal wash of LAIV/Spn- (*n=37*) and LAIV/Spn+ (*n=43*)**. D) I**nfluenza-specific IgM titres in TIV (n=20), LAIV (n=19) vaccinated subjects and unvaccinated (n=12) measured by ELISA in

BAL fluid. **E)** Influenza-specific IgM titres in BAL grouped based on vaccination and colonization status, as TIV/Spn- (*n=9*), TIV/Spn+ (*n=11*), LAIV/Spn- (*n=11*), LAIV/ Spn+ (*n=8*), unvaccinated (n=12). Geometric means with 95% CI are shown.* p <0.05, ** p<0.01, ***p<0.001 **** p < 0.0001 by Wilcoxon test for comparisons within the same group and by Mann-Whitney test for comparisons between groups.

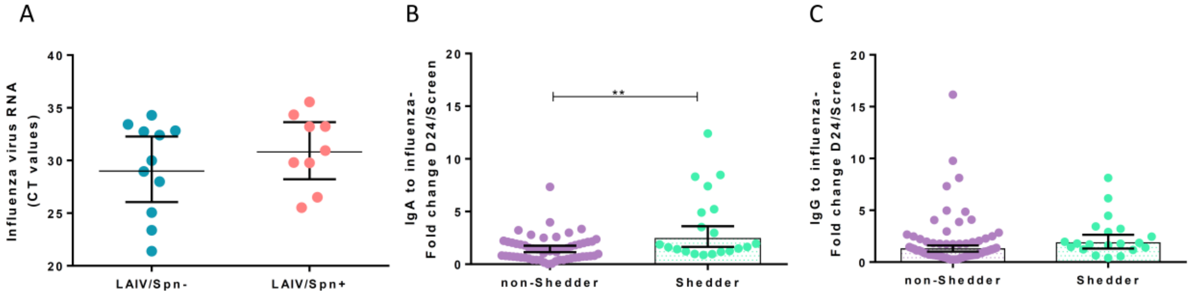

Supplementary figure 7: Live attenuated influenza virus replication in the nasopharynx elicits influenza virus specific-IgA production in the local mucosa. (A) Levels of detectable influenza RNA (CT values) 3 days post LAIV administration in both LAIV/Spn- (n=11) and LAIV/Spn+ (n=9). Fold change (D24/Baseline) of paired (B) IgA and (C) IgG titres to influenza in nasal wash of LAIV vaccinated volunteers. Subjects were grouped in influenza virus non-shedders (*n=59*) or shedders (*n=20*) ** p < 0.002 by Mann-Whitney test. Each individual dot represents a single volunteer and geometric means with 95% CI are shown.

	**LAIV vaccinated**		
**Colonization status**	**Shedders**		**Non-Shedders**
	**Influenza A**	**Influenza B**	
**Carriage negative**	3	8	29
**Carriage positive**	1	8	30
**Percentage**	5.0%	20.3%	74.6%

Supplementary Table 1: Influenza virus shedding in the LAIV vaccinated group. Virus shedding (either influenza A or influenza B strains) was detected in 25.3% (20/79) of the LAIV vaccinated group, by utilizing RNA qPCR on nasal wash samples collected at D3. Colonization status did not affect influenza strains replication in the nasopharynx. p > 0.05 by Fisher's exact test.
